# Psychological trauma as a transdiagnostic risk factor for mental disorder: an umbrella meta‐analysis

**DOI:** 10.1192/j.eurpsy.2023.2089

**Published:** 2023-07-19

**Authors:** B. M. Hogg, I. Gardoki-Souto, A. Valiente-Gómez, A. Ribeiro Rosa, L. Fortea, J. Radua, B. L. Amann, A. Moreno-Alcázar

**Affiliations:** 1 Hospital del Mar Medical Research Institute, Barcelona; 2 Department of Psychiatry and Legal Medicine, Universitat Autonòma de Barcelona, Bellaterra; 3 Centre Fòrum Research Unit, Institute of Neuropsychiatry and Addiction (INAD), Parc de Salut Mar, Barcelona; 4 Centro de Investigación Biomédica en Red de Salut Mental (CIBERSAM), Madrid; 5 Centre Fòrum; 6 Hospital del Mar Research Institute, Barcelona, Spain; 7 Laboratory of Molecular Psychiatry, Hospital de Clínicas de Porto Alegre; 8 Universidade Federal do Rio Grande do Sul, Porto Alegre, Brazil; 9 Imaging of Mood- and Anxiety-Related Disorders (IMARD) group, Institut d’Investigacions Biomèdiques August Pi i Sunyer (IDIBAPS); 10 Institute of Neurosciences, University of Barcelona, Barcelona, Spain; 11 Department of Psychosis Studies, King’s College London, London, United Kingdom; 12 Department of Clinical Neuroscience, Karolinska Institute, Stockholm, Sweden; 13 Universitat Pompeu Fabra, Barcelona, Spain; 14 Clinic for Psychiatry and Psychotherapy, Klinikum der Universität München, Munich, Germany; 15 Centre Fòrum Research Unit, Hospital del Mar Research Institute, Barcelona, Spain

## Abstract

**Introduction:**

This umbrella review is the frst to systematically examine psychological trauma as a transdiagnostic risk factor across psychiatric conditions.

**Objectives:**

This review aimed to be the frst to evaluate whether psychological trauma fulflilled criteria as a transdiagnostic risk factor cutting across various diagnostic categories and spectra. Transdiagnosticity will be assessed against the framework of the TRANSD criteria (Fusar-Poli, World Psychiatry 2019; 18 361-362). The paper additionally aimed to analyse the association of psychopathology with specifc trauma type.

**Methods:**

We searched Pubmed, Scopus, and PsycNET databases from inception until 01/05/2021 for systematic reviews/meta-analyses evaluating the association between psychological trauma and at least one diagnosed mental disorder. We re-calculated the odds ratio (OR), then classifed the association as convincing, highly suggestive, suggestive, or weak, based on the number of cases and controls with and without psychological trauma, random-efects p value, the 95% conf- dence interval of the largest study, heterogeneity between studies, 95% prediction interval, small-study efect, and excess significance bias. Additional outcomes were the association between specifc trauma types and specific mental disorders, and a sensitivity analysis for childhood trauma. Transdiagnosticity was assessed using TRANSD criteria. The review was pre-registered in Prospero CRD42020157308 and followed PRISMA/MOOSE guidelines.

**Results:**

Fourteen reviews met inclusion criteria, comprising 16,277 cases and 77,586 controls. Psychological trauma met TRANSD criteria as a transdiagnostic factor across diferent diagnostic criteria and spectra. There was highly suggestive evidence of an association between psychological trauma at any time-point and any mental disorder (OR=2.92) and between childhood trauma and any mental disorder

(OR=2.90). Regarding specifc trauma types, convincing evidence linked physical abuse (OR=2.36) and highly suggestive evidence linked sexual abuse (OR=3.47) with a range of mental disorders, and convincing evidence linked emotional abuse to anxiety disorders (OR=3.05); there were no data for emotional abuse with other disorders.

**Image:**

**Figure:**
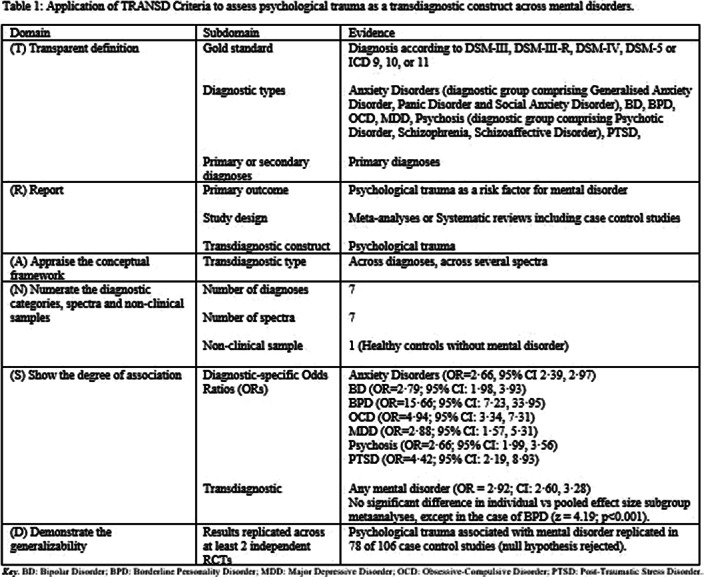

**Image 2:**

**Figure:**
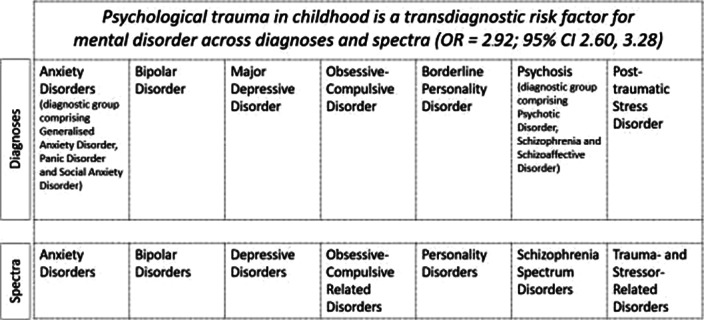

**Conclusions:**

These fndings highlight the importance of preventing early traumatic events and providing trauma-informed care in early intervention and psychiatric services.

**Disclosure of Interest:**

None Declared

